# Contrast enhanced ultrasound combined with serology predicts hepatocellular carcinoma recurrence: a retrospective observation cohort study

**DOI:** 10.3389/fonc.2023.1154064

**Published:** 2023-07-14

**Authors:** Haibin Tu, Siyi Feng, Lihong Chen, Yujie Huang, Juzhen Zhang, Xiaoxiong Wu

**Affiliations:** ^1^ Department of Ultrasound, Mengchao Hepatobiliary Hospital of Fujian Medical University, Fuzhou, Fujian, China; ^2^ Department of Oncology, Seventh People’s Hospital of Shanghai University of Traditional Chinese Medicine, Shanghai, China

**Keywords:** hepatocellular carcinoma, contrast-enhanced ultrasound, early recurrence, prediction, serological markers

## Abstract

**Objectives:**

To construct a novel model based on contrast-enhanced ultrasound (CEUS) and serological biomarkers to predict the early recurrence (ER) of primary hepatocellular carcinoma within 2 years after hepatectomy.

**Methods:**

A total of 466 patients who underwent CEUS and curative resection between 2016.1.1 and 2019.1.1 were retrospectively recruited from one institution. The training and testing cohorts comprised 326 and 140 patients, respectively. Data on general characteristics, CEUS Liver Imaging Reporting and Data System (LI-RADS) parameters, and serological were collected. Univariate analysis and multivariate Cox proportional hazards regression model were used to evaluate the independent prognostic factors for tumor recurrence, and the Contrast-enhanced Ultrasound Serological (CEUSS) model was constructed. Different models were compared using prediction error and time-dependent area under the receiver operating characteristic curve (AUC). The CEUSS model's performances in ER prediction were assessed.

**Results:**

The baseline data of the training and testing cohorts were equal. LI-RADS category, α-fetoprotein level, tumor maximum diameter, total bilirubin level, starting time, iso-time, and enhancement pattern were independent hazards, and their hazards ratios were 1.417, 1.309, 1.133, 1.036, 0.883, 0.985, and 0.70, respectively. The AUCs of CEUSS, BCLC,TNM, and CNLC were 0.706, 0.641, 0.647, and 0.636, respectively, in the training cohort and 0.680, 0.583, 0.607, and 0.597, respectively, in the testing cohort. The prediction errors of CEUSS, BCLC, TNM, and CNLC were 0.202, 0.205, 0.205, and 0.200, respectively, in the training cohort and 0.204, 0.221, 0.219, and 0.211, respectively, in the testing cohort.

**Conclusions:**

The CEUSS model can accurately and individually predict ER before surgery and may represent a new tool for individualized treatment.

## Introduction

Liver cancer has the sixth highest global incidence and the third highest mortality rate, dominantly hepatocellular carcinoma (HCC) (1). Transplantation has been used as an effective treatment modality for HCC in developed countries for many years. Given the shortage of liver sources and the high cost of treatment, liver transplantation is not usually performed in developing countries. Moreover, when tumors are detected in most patients, their conditions are beyond the criteria for transplantation; therefore, currently, surgery remains one of the most effective treatment modalities as it is effective in improving the survival rate of most patients. However, postoperative recurrence, particularly within 2 years postoperatively, is defined as early recurrence (ER) and is still an important hazard that reduces the survival rate (2–6). If a model that can identify patients at high risk of ER can be created, more appropriate treatments and adjuvant therapy can be available for improving the prognosis (7, 8).

Nowadays, widely used tumor grading systems, such as the tumor lymph node metastasis (TNM), Barcelona Clinical Liver Cancer (BCLC) staging, and China Liver Cancer Staging (CNLC), are essential for guiding treatment; however, their value in predicting recurrence needs further investigation. Although there are predictive models available for postoperative recurrence, they are created based on postoperative pathological findings. If an ER prediction model can be created based on preoperative markers, it will be of great aid for personalized treatment. Moreover, neoadjuvant therapy can improve the prognosis of patients (9–12).

Owing to the development of radiological techniques and serological detection capabilities, noninvasive prediction of postoperative recurrence has become possible. Although radiology techniques (artificial intelligence, radiomics) or serologic markers (mortalin, nucleolar, and spindle-associated protein 1) are used to predict recurrence (13–16), artificial intelligence and radiomics are complex and difficult to master and serologic markers change dynamically. Studies combining the two techniques are extremely rare. Ultrasound is chosen by clinicians because of its noninvasiveness, reproducibility, and affordability. Among them, contrast-enhanced ultrasound (CEUS) can observe blood perfusion of tumors in real-time and has unique advantages for determining the nature of tumors.

In the present study, CEUS was combined with serological markers to create a noninvasive prediction model, called Contrast-enhanced Ultrasound Serological (CEUSS) model. The predictive accuracy of this model was validated using an internal independent validation cohort. Our model was compared with other tumor staging systems, such as TNM, BCLC, and CNLC. We believe that this can be an effective model that can help screen the high-risk recurrence population.

## Materials and methods

### Patient population

This study was approved by the Ethics Committee and Institutional Review Board of Mengchao Hepatobiliary Hospital of Fujian Medical University, China (Approval No. 2020-010-01). All patients were informed in writing of the study protocol and objectives.

In total, 1320 patients who met the inclusion criteria and underwent curative resection at Mengchao Hepatobiliary Hospital between January 1, 2016, and January 1, 2019, were retrospectively recruited.

The inclusion criteria were as follows: (1) age: 18–70 years, male or female; (2) Liver function: Child–Pugh A (3) indocyanine green 15-min retention test: <15%; (4) pathologically confirmed HCC and achievement of R0 resection; and (5) CEUS was performed preoperatively.

**Figure 1:**
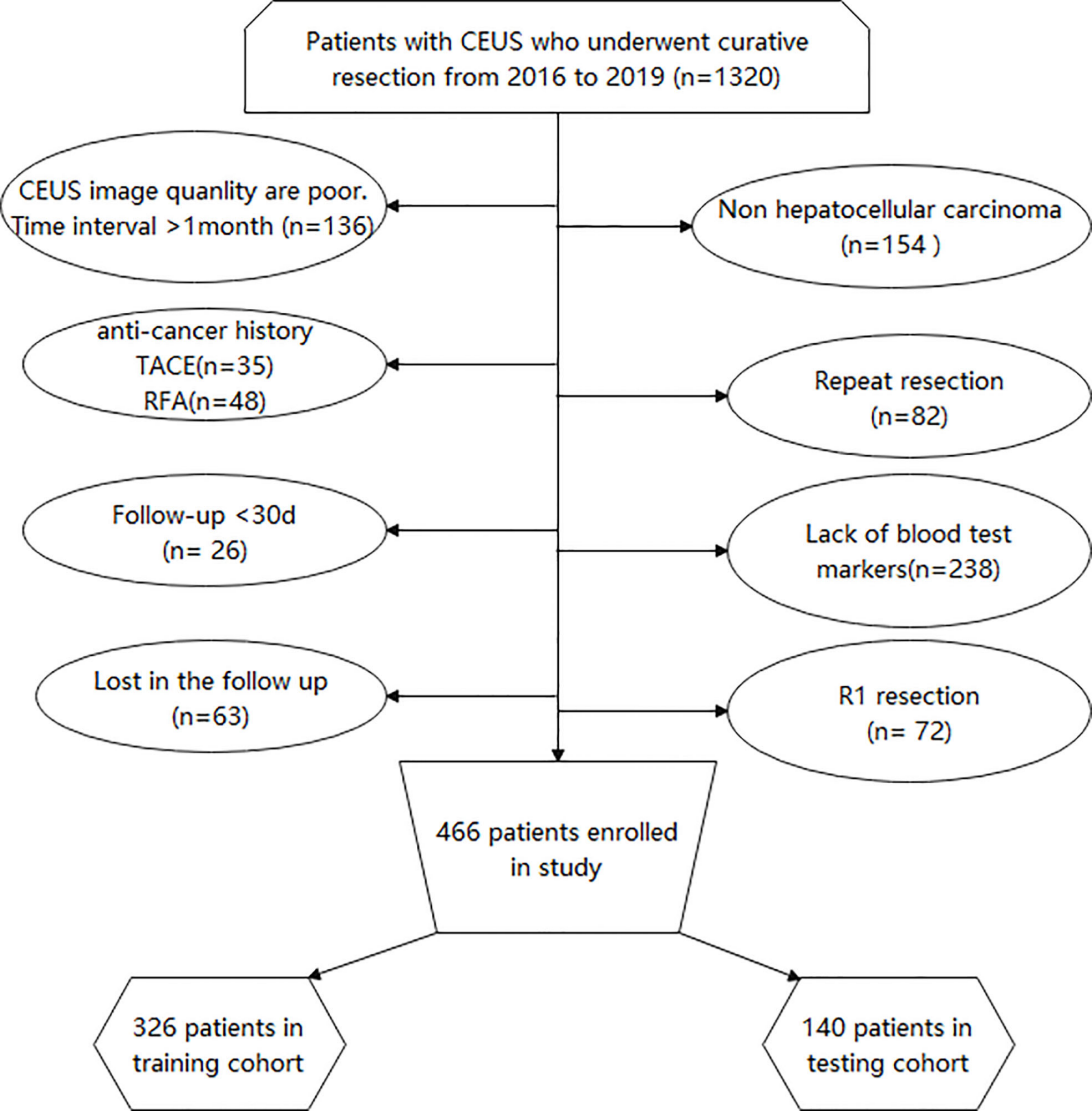
Flow program for patient inclusion. CEUS, Contrast-enhanced ultrasound; TACE, Transcatheter arterial chemoembolization; RFA, Radiofrequency ablation.

The exclusion criteria were as follows: (1) patients with postoperative pathology confirmed as nonhepatocellular carcinoma including cholangiocarcinoma and combined hepatocellular-cholangiocarcinoma; (2) those with unclear ultrasound images; (3) time between CEUS and surgery: >1 month; (3) those who underwent other treatments including radiotherapy and chemotherapy; (4) those who underwent palliative surgery or repeated resection; and (5) those with incomplete clinical data or who were lost to follow-up. Finally, 446 patients were enrolled in this study (**Figure 1**).

### CEUS technique

**Figure 2:**
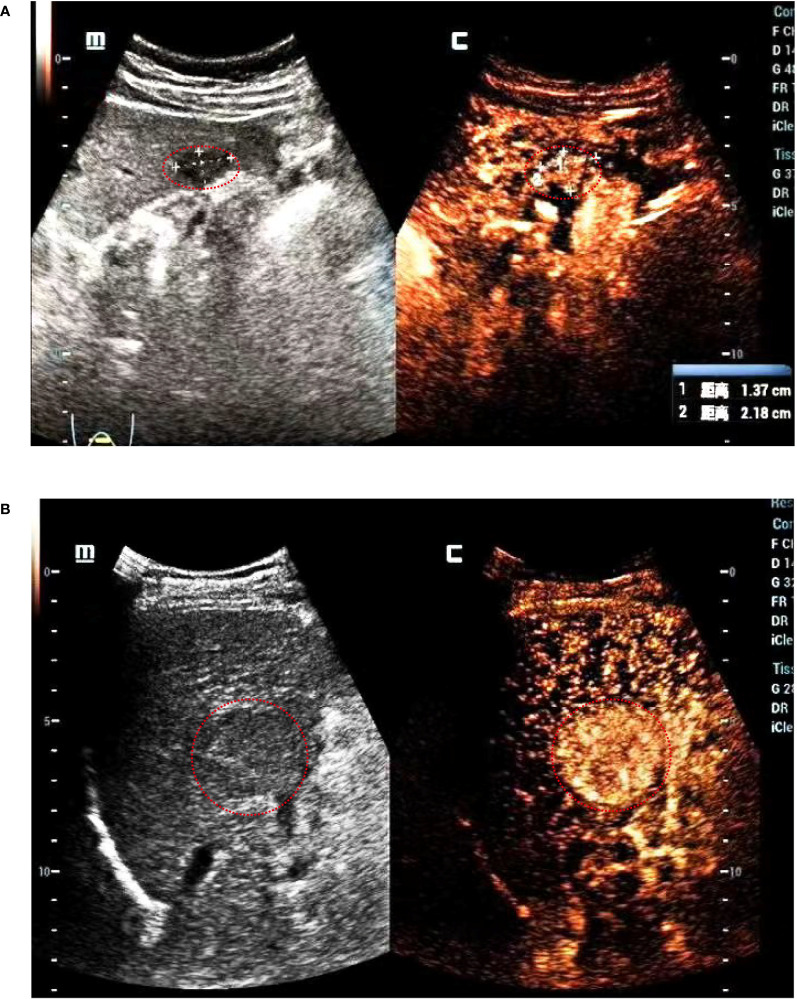
LI-RADS shows that **(A)** the tumor diameter is <2 cm, it is defined as LI-RADS 4. **(B)** The tumor diameter is 4.3 cm, it is defined as LI-RADS 5.

CEUS was performed in all patients with Italian Esaote ultrasound machines using C5-2 convex array probes with Sonovue (Bracco, Milan, Italy) as the contrast agent, operated by ultrasonographers (Jianlin Lin) with very high experience in CEUS examinations. Next, 2.4 mL SonoVue was rapidly injected through an antecubital vein followed by a 10 mL saline flush. A sufficiently large needle (minimum diameter: 20 gauge) should be used to avoid causing bubble ruptures. A low mechanical index (<0.1) was used for the CEUS examination. The ultrasound contrast images were stored in an external system in the DICOM format. Two senior ultrasound physicians with over 10 years of experience (Haibin Tu and Siyi Feng) analyzed the tumor B mode and contrast images together and determined the Liver Imaging Reporting and Data System (LI-RADS) classification of the tumor. In cases where the two physicians could not reach a consensus, a third ultrasound physician (Jianlin Lin) with 25 years of work experience was then invited to discuss the case together and determine the LI-RADS category of the tumor. All three doctors assessed the images without knowledge of the pathological findings. The CEUS terminology follows the American College of Radiology CEUS LI-RADS working group standard (17). LI-RADS 4 and LI-RADS 5 are shown in **Figure 2**. LI-RADS TIV was defined as 6. The following parameters were also collected: (a) the maximum tumor diameter, (b) starting time: the time from the injection to entering the tumor, (c) peak time: the time from the injection to the maximum intensity of the tumor, (d) iso-time: the time from the injection to the tumor enhancement was equal to peripheral, (e) washout time: the time from the injection to the tumor enhancement lower than peripheral, (f) enhancement type: fast-in fast-out and non-fast-in fast-out, (g) wash-in type, after contrast agent injection, if the tumor perfusion was faster than the peripheral tissues, it is defined as fast in; otherwise, it is defined as non-fast-in, (h) wash-out type: If the tumor washes out faster than the peripheral tissue 30 s after contrast agent injection, it is defined as fast-out; otherwise, it is defined as non-fast-out, (i) enhancement echogenicity: homogeneous and inhomogeneous, and (j) tumor location: half liver, and non-half-liver, half liver means tumor is located only in the left or right liver.

### Serological markers

The fasting serological measurements within 3 days before treatment were collected through the medical records. The serological markers included serum aspartate aminotransferase, alanine aminotransferase, platelet, albumin, total bilirubin (TBIL), and creatinine levels; prothrombin time; international normalized ratio; and prothrombin time activity. The serum alpha-fetoprotein (AFP) level was divided into three categories: 0–20, 20–200, and >200 μg/L.

### Follow-up surveillance

Following the surgical resection, patients underwent regular follow-up assessments for screening HCC recurrence, including monitoring of serum AFP levels and conducting liver function tests, abdominal ultrasound, and contrast-enhanced computed tomography (CT) or magnetic resonance imaging (MRI) scans of the chest and abdomen during the first month and then at three-month intervals until 2 years. Tumor recurrence was defined as the appearance of new lesions with imaging features typical of HCC, diagnosed using at least two imaging modalities and reported by two independent radiologists blinded to the group assignment. All data were evaluated in October 2021.

### Staging system

The TNM staging system was proposed in 2017 (18). The TNM stages Ia and Ib were classified into one category. The BCLC staging system was proposed in 2021 (19). BCLC0 and BCLC A were classified into one category. The CNLC staging system was proposed in 2019. The CNLC stages Ia and Ib were classified into one category, stages IIa and IIb into one category, and stages IIIa and IIIb into one category.

### Statistical analysis

The patients were randomly divided into training and testing cohorts in a 7:3 ratio. Categorical variables were presented as percentages and compared using chi-square tests. Continuous variables that met normality were tested using student’s t-test (two-sided), otherwise Mann–Whitney U test was used. The Kaplan–Meier (KM) curves were compared between the high- and low-recurrence risk groups based on log-rank. The inclusion of predictors was guided by the following factors: previous literature reports, differences in univariate analysis, and clinical significance.

All variables were evaluated using a correlation matrix to verify the presence of collinearity. LI-RADS, wash-out time, and wash-out type had collinearity. LI-RADS was selected for its maximum hazard ratio (HR). We tested whether the variables, including age, sex, tumor size, and LI-RADS, had internal interactions and found no obvious interaction. The association between clinical markers and recurrence-free survival was assessed using the Cox proportional hazards regression model. The backward method was used to calculate the model to find the best predictor. HR was used to represent the effect of each index on the risk of ER, and their 95% confidence interval (CI) is presented. Considering both clinical practicalities, the LI-RADS category, serum AFP level, and tumor diameter were finally selected. Nomograms were constructed using the selected variables and were used to predict ER. After the nomogram was constructed, the C-index was used to describe the accuracy of the model and calibration curves were plotted to describe the discriminatory ability of the model. To find the best cutoff value for clinical use, X-tile (version 3.6.1) was used. The prediction error values for CEUSS and other tumor grading systems were evaluated over 2 years using the “Boot632plus” split method (20). Time-dependent receiver operating characteristic (ROC) curves and the areas under the ROC curve (AUCs) were drawn. Statistical analyses were performed using R software version 3.6.3 (http://www.r-project.org). All tests were two-sided, and P < 0.05 was considered statistically significant (20).

## Results

### Baseline data

In total, 466 patients were included (**Figure 1**): 326 patients in the training cohort and 140 patients in the testing cohort. The two cohorts were equal at baseline (**Table 1**).

**Table 1:** Clinical characteristics of the patients.

	Total	train	test	P-value
censor				
non-recurrence	270 (57.9%)	195 (59.8%)	75 (53.6%)	0.22
recurrence	196 (42.1%)	131 (40.2%)	65 (46.4%)	
Diameter(cm)	3.2[2.2,4.5]	3.3[2.3,4.6]	3.0[2.2,4.8]	0.61
Age(years)	56.0±11.3	55.8±11.5	56.6±10.9	0.45
ALT(U/L)	35.9±29.9	36.3±30.4	35.0±28.7	0.69
AST(U/L)	35.2±31.3	34.6±31.3	36.5±31.2	0.35
PLT(/L)	154.0±69.5	156.1±70.2	149.0±67.9	0.29
ALB(g/L)	39.2±4.5	39.1±4.6	39.2±4.4	0.78
TBIL(umol/L)	16.8±8.4	16.7±8.7	17.2±7.8	0.24
Cr(umol/L)	74.3±17.8	74.1±17.3	74.8±19.0	0.94
PT(s)	13.6±1.2	13.6±1.3	13.5±1.0	0.47
PTA(%)	95.3±16.2	95.0±17.0	96.2±14.2	0.38
INR	1.0±0.1	1.1±0.1	1.0±0.1	0.38
Initial enhanced time(s)	16.7±3.6	16.7±3.8	16.6±3.2	0.98
Time to peak(s)	23.8±5.5	24.0±5.8	23.3±4.7	0.5
Time to iso-enhanced(s)	40.2±15.5	40.6±16.8	39.2±11.7	0.95
Washout time(s)	100.0±63.1	99.4±64.9	101.3±58.9	0.4
Cirrhosis				
no	266(57.1%)	184(56.4%)	82(58.6%)	0.81
yes	200(42.9%)	142(43.6%)	58(41.4%)	
Etiology				
Hepatitis B	409(87.8%)	287(88.0%)	122(87.1%)	0.85
Hepatitis C	15(3.2%)	10(3.1%)	5(3.6%)	
Other	42(9.0%)	29(8.9%)	13(9.3%)	
Sex				
Male	387 (83.0%)	272 (83.4%)	115 (82.1%)	0.79
Female	79 (17.0%)	54 (16.6%)	25 (17.9%)	
LIRADS				
3	14 (3.0%)	10 (3.1%)	4 (2.9%)	0.07
4	179 (38.4%)	129 (39.6%)	50 (35.7%)	
5	262 (56.2%)	180 (55.2%)	82 (58.6%)	
6	11 (2.4%)	7 (2.1%)	4 (2.8%)	
AFP(ug/L)				
<20	251 (53.9%)	180 (55.2%)	71(50.7%)	0.48
20-200	78 (16.7%)	51 (15.6%)	27(19.3%)	
>200	137 (29.4%)	95 (29.1%)	42(30.0%)	
Fast in fast out				
no	51 (10.9%)	37 (11.3%)	14(10.0%)	0.52
yes	415 (89.1%)	289 (88.7%)	126(90.0%)	
Fast in				
no	5 (0.1%)	4 (1.2%)	1 (0.7%)	1
yes	461 (99.9%)	322 (98.8%)	139(99.3%)	
Fast out				
no	129 (27.7%)	82 (25.2%)	47(33.6%)	0.45
yes	337 (72.3%)	244 (74.8%)	93(66.4%)	
Homogeneous enhancement				
yes	310 (66.5%)	211 (64.7%)	99(70.7%)	0.85
no	156 (33.5%)	115 (35.3%)	41(29.3%)	
Only located in left or right lobe of liver				
yes	412 (88.4%)	291 (89.3%)	121 (86.4%)	0.43
no	54 (11.6%)	35 (10.7%)	19 (13.6%)	
TNM				
Ia,Ib	314 (67.4%)	219 (67.2%)	95(67.9%)	0.75
II	103 (22.1%)	74 (22.7%)	29(20.7%)	
III	49 (10.5%)	33 (10.1%)	16(11.4%)	
BCLC				
A1-A4	392 (84.1%)	274 (84.0%)	118(84.2%)	0.8
B	49 (10.5%)	38 (11.7%)	11(7.9%)	
C	25 (5.4%)	14 (4.3%)	11(7.9%)	
CNLC				
Ia,Ib	396 (85.0%)	276 (84.7%)	117 (87.2%)	0.82
IIa,IIb	42 (9.0%)	33 (10.1%)	9(6.4%)	
IIIa,IIIb	28 (6.0%)	17 (5.2%)	9(6.4%)	

ALT, Alanine aminotransferase; AST, Aspartate aminotransferase; PLT, Platelet; AFP, Alpha-fetoprotein; ALB, Albumin; TBIL, Total bilirubin; Cr, Serum creatinine; PT, Prothrombin time; PTA, Prothrombin time activity; INR, International normalized ratio; LIRADS, Liver imaging reporting and data system.

In the training cohort, the following variables differed between the recurrence and no-recurrence groups: LI-RADS category, serum AFP level, maximum tumor diameter, TBIL, washout time, and TNM, BCLC, and CNLC stages (**Table 2**). The backward Cox regression method was used to calculate the indexes that might be important for predicting ER; **Table 3** shows the results. The independent predictive factors for ER were the LI-RADS category, serum AFP level, tumor diameter, TBIL, start time, iso-time, and enhancement type.

**Table 2:** Clinical characteristics of the training cohort.

	Total	non-recurrence	recurrence	P-value
Diameter(cm)	3.3[2.3,4.6]	2.7[2.0,3.5]	4.0[2.7,5.8]	< 0.001
Age(years)	55.8±11.5	56.4±11.2	55.8±10.8	0.51
ALT(U/L)	36.3±30.4	33.8±23.6	39.8±38.7	0.2
AST(U/L)	34.6±31.3	31.6±18.1	39.2±40.7	0.26
PLT(/L)	156.1±70.2	160.4±69.0	149.1±67.5	0.13
ALB(g/L)	39.1±4.6	39.4±4.9	39.1±4.3	0.39
TBIL(umol/L)	16.7±8.7	15.9±7.9	17.5±9.0	0.19
Cr(umol/L)	74.1±17.3	75.0±21.4	74.1±13.1	0.52
PT(s)	13.6±1.3	13.5±1.2	13.6±1.3	0.89
PTA(%)	95.0±17.0	96.5±16.6	95.4±16.8	0.86
INR	1.1±0.1	1.0±0.1	1.1±0.1	0.78
Initial enhanced time(s)	16.7±3.8	17.1±3.8	16.4±3.2	0.14
Time to peak(s)	24.0±5.8	24.1±5.6	23.5±5.5	0.25
Time to iso-enhanced(s)	40.6±16.8	40.3±13.2	38.6±13.2	0.13
Washout time(s)	99.4±64.9	99.7±58.3	88.4±62.0	0.025
Cirrhosis				
no	184(56.4%)	113(57.9%)	71(54.2%)	0.57
yes	142(43.6%)	82(42.1)	60(45.8%)	
**Etiology**				
Hepatitis B	287(88.0%)	169(86.7%)	118(90.1%)	0.65
Hepatitis C	10(3.1%)	6(3.1%)	4(3.0%)	
Other	29(8.9%)	20(10.2)	9(6.9%)	
Sex				
Male	272 (83.4%)	156 (80.0%)	116 (88.5%)	0.054
Female	54 (16.6%)	39 (20.0%)	15 (11.5%)	
LIRADS				
3	10 (3.1%)	8 (4.1%)	2 (1.5%)	0.03
4	129 (39.6%)	86 (44.1%)	43 (32.8%)	
5	180 (55.2%)	99 (50.8%)	81 (61.8%)	
6	7 (2.1%)	2 (1.0%)	5 (3.8%)	
AFP(ug/L)				
<20	180 (55.2%)	127 (65.1%)	53 (40.5%)	< 0.001
20-200	51 (15.6%)	29 (14.9%)	22 (16.8%)	
>200	95 (29.1%)	39 (20.0%)	56 (42.7%)	
Fast in fast out				
no	37 (11.3%)	17 (8.7%)	20 (15.3%)	0.076
yes	289 (88.7%)	178 (91.3%)	111 (84.7%)	
Fast in				
no	4 (1.2%)	2 (1.0%)	2 (1.5%)	1
yes	322 (98.8%)	193 (99.0%)	129 (98.5%)	
Fast out				
no	82 (25.2%)	48 (24.6%)	34 (26.0%)	0.8
yes	244 (74.8%)	147 (75.4%)	97 (74.0%)	
Homogeneous enhancement				
yes	211 (64.7%)	121 (62.1%)	90 (68.7%)	0.24
no	115 (35.3%)	74 (37.9%)	41 (31.3%)	
Only located in left or right lobe of liver				
yes	291 (89.3%)	179 (91.8%)	112 (85.5%)	0.099
no	35 (10.7%)	16 (8.2%)	19 (14.5%)	
TNM				
Ia,Ib	219 (67.2%)	150 (76.9%)	69 (52.7%)	< 0.001
II	74 (22.7%)	38 (19.5%)	36 (27.5%)	
III	33 (10.1%)	7 (3.6%)	26 (19.8%)	
BCLC				
A1-A4	274 (84.0%)	184 (94.4%)	90 (68.7%)	< 0.001
B	38 (11.7%)	10 (5.1%)	28 (21.4%)	
C	14 (4.3%)	1 (0.5%)	13 (9.9%)	
CNLC				
Ia,Ib	276 (84.7%)	185 (94.9%)	91 (69.5%)	< 0.001
IIa,IIb	33 (10.1%)	9 (4.6%)	24 (18.3%)	
IIIa,IIIb	17 (5.2%)	1 (0.5%)	16 (12.2%)	

ALT, Alanine aminotransferase; AST, Aspartate aminotransferase; PLT, Platelet; AFP, Alpha-fetoprotein; ALB, Albumin; TBIL, Total bilirubin; Cr, Serum creatinine; PT, Prothrombin time; PTA, Prothrombin time activity; INR, International normalized ratio; LIRADS, Liver imaging reporting and data system.

**Table 3:** Analysis of the prognostic factors affecting the early recurrence.

	β	P value	OR	95.0% CI	
				Lower	Upper
LI-RADS	0.348	0.046	1.417	1.006	1.995
AFP	0.269	0.012	1.309	1.061	1.614
Diameter	0.125	0.000	1.133	1.062	1.208
TBIL	0.036	0.002	1.036	1.013	1.060
PT	-0.473	0.073	0.623	0.372	1.045
PTA	-0.042	0.055	0.959	0.919	1.001
Initial enhanced time	-0.125	0.000	0.883	0.825	0.944
Time to iso-enhanced	-0.015	0.038	0.985	0.971	0.999
homogeneous enhancement	-0.357	0.077	0.700	0.471	1.039

LIRADS, Liver imaging reporting and data system; AFP, Alpha-fetoprotein; TBIL, Total bilirubin; PT, Prothrombin time; PTA, Prothrombin time activity; HR, Hazard ratio; CI, Confidence interval.

### Nomogram construction

**Figure 3:**
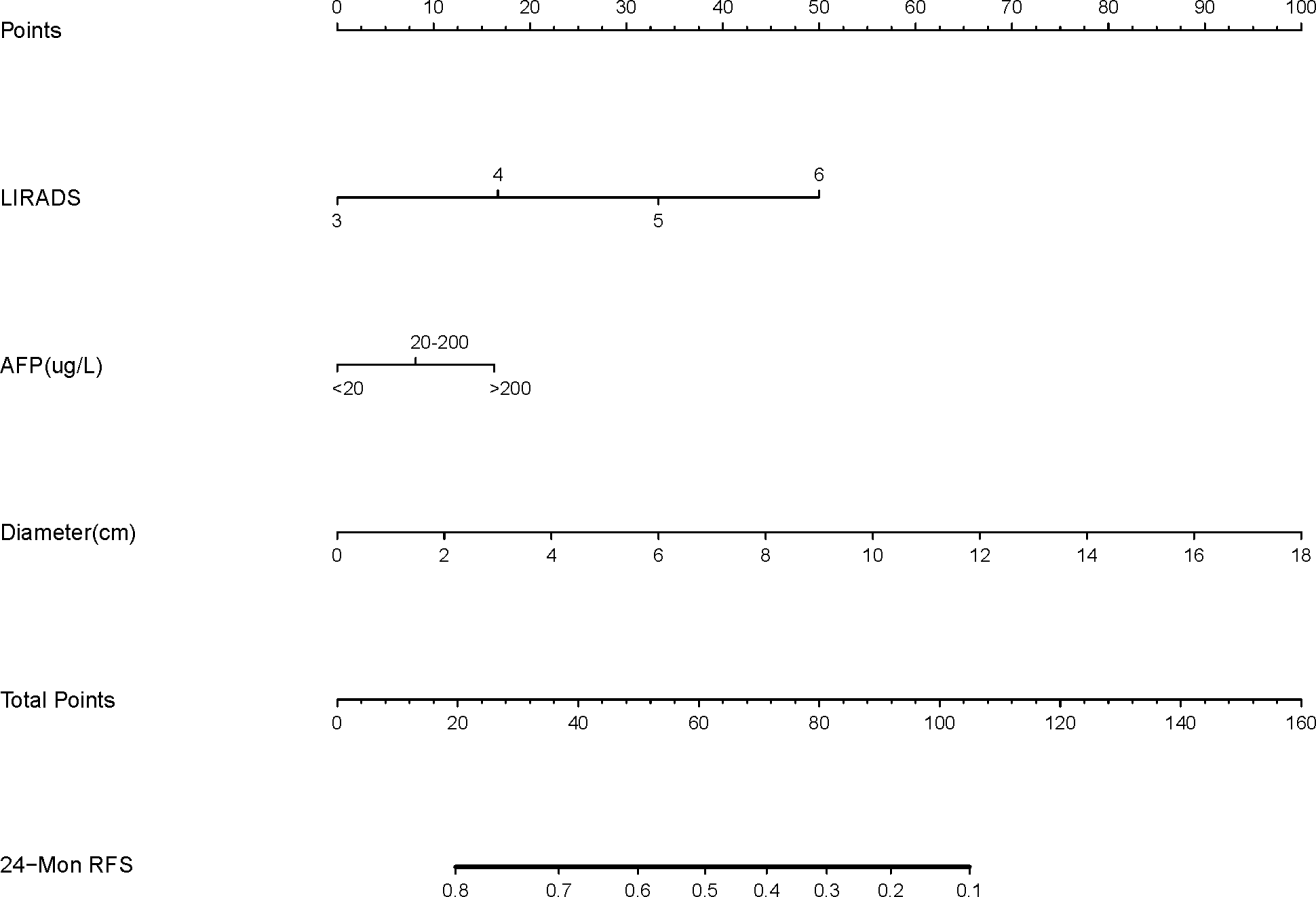
Nomogram structure based on the LI-RADS category, AFP level, and tumor diameter. LI-RADS, Liver Imaging Reporting and Data System; AFP, Alpha-fetoprotein; RFS, Recurrence-free survival.

LI-RADS, serum AFP level, and maximum tumor diameter were finally selected as the basic factors for the nomograms and the CEUSS model by excluding collinearity and selecting simple markers. The nomograms are shown in **Figure 3**.

### Prediction error

**Figure 4:**
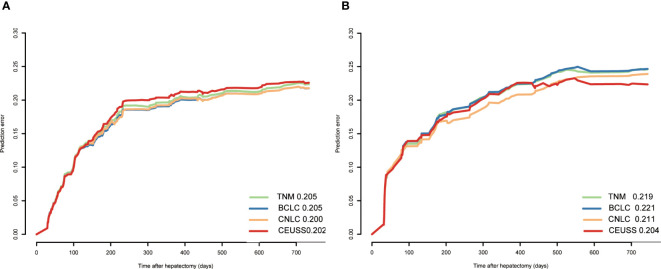
Prediction error of different models in the **(A)** training cohort and **(B)** testing cohort. CEUSS, Contrast-enhanced ultrasound serological.

The prediction error plots of different tumor staging systems were drawn (**Figure 4**), and prediction error values were calculated (**Table 4**). The prediction error value of the CEUSS model was slightly lower than that of the other models, with no statistically significant difference.

**Table 4:** Predict error and td-AUC of models in training and testing cohort.

	predict error		time dependent AUC(95%CI)	
	train	test	train	test
TNM	0.205	0.219	0.647(0.594,0.701)	0.607(0.529,0.685)
BCLC	0.205	0.221	0.641(0.598,0.685)	0.583(0.522,0.644)
CNLC	0.200	0.211	0.636(0.594,0.679)	0.597(0.538,0.656)
CEUSS	0.202	0.204	0.706(0.648,0.764)	0.680(0.592,0.769)

AUC, Area under the curve; CEUSS, Contrast-enhanced ultrasound serological.

### Time-dependent area under the ROC curve

**Figure 5:**
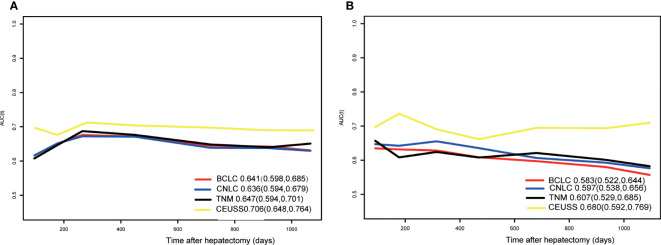
Time-dependent ROC curves in the **(A)** training cohort and **(B)** testing cohort. ROC, receiver operating characteristic.

The time-dependent area under the ROC curve plots of the models was drawn (**Figure 5**), and their AUCs were calculated (**Table 4**). The AUC of CEUSS was higher than that of the other models.

### Calibration curve

**Figure 6:**
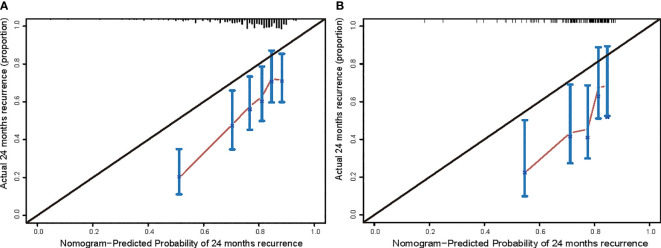
Calibration curves in the **(A)** training cohort and **(B)** testing cohort.

The calibration curve was drawn between the training and testing cohorts according to the CEUSS model (**Figure 6**). The coincidence degree between the predicted and real values was satisfactory.

### KM curve

**Figure 7:**
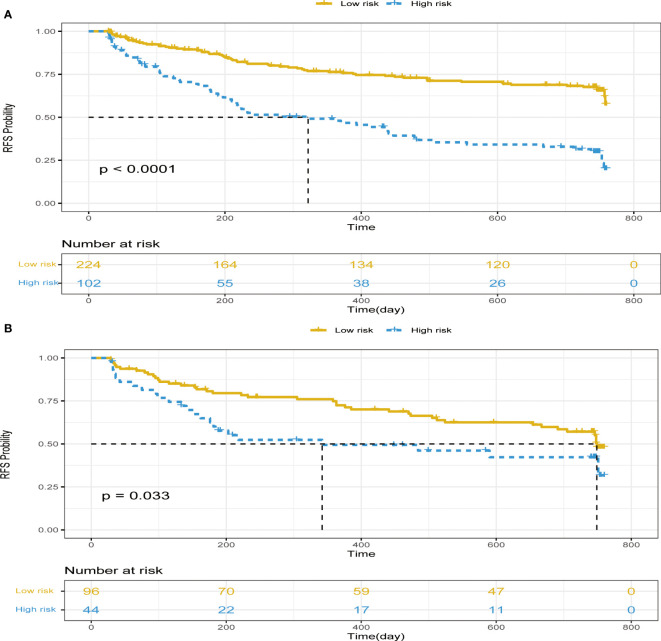
KM curves in the **(A)** training cohort and **(B)** testing cohort. KM, Kaplan–Meier.

The CEUSS model was used for calculating the risk score of each patient, and the patients were classified into low- and high-risk recurrence groups with 50 as the cutoff value. The KM curve between both cohorts was drawn (**Figure 7**). A considerable difference was observed between the high- and low-recurrence risk groups.

## Discussion

We herein created and validated a noninvasive prediction model called the CEUSS model based on CEUS and serological markers for HCC. The CEUSS model showed satisfactory prediction results with C indexes of 0.71 (95% CI: 0.65–0.76) in the training cohort and 0.68 (95% CI: 0.59–0.77) in the testing cohort, which were higher than those of other tumor staging systems. The model was able to accurately distinguish the patients at high risk for ER from the training and testing cohorts.

Although many methods are currently available for the treatment of HCC, such as liver transplantation, radiofrequency, transcatheter arterial chemoembolization, and radioactive particle therapy (21), surgical resection remains the main method of treatment; however, postoperative recurrence, particularly ER, severely worsens the prognosis of HCC. ER rates of up to 30%–50% have been reported previously (22, 23), which is similar to the findings of our study, wherein the rate was 42.1%. If the high-recurrence risk group can be screened early and provided positive treatment preoperatively, such as immunotherapy, target therapy, transcatheter arterial chemoembolization, and neoadjuvant chemotherapy (9–12), the treatment outcome can be improved. Previously, CT, MRI, positron emission tomography, genetic markers, and serological markers have been used for predicting ER, and some prediction models have been created; however, some of these markers are not universal, such as radiomics. Ultrasound is a routine method for liver cancer screening, and CEUS has many advantages in identifying malignant tumors; therefore, in this study, CEUS was chosen, and the less commonly used markers were discarded. Only universal markers were used as modeling factors to ensure that the model could be replicated in clinical practice. In addition to the CEUS indexes, the maximum tumor diameter and serum AFP level, which are two commonly used indexes, were also included in the final model to ensure the clinical usefulness of the model.

Unlike CT and MRI contrast agents, an ultrasound contrast agent is a blood sinus type that can observe tumor microvasculature in real time. Tumors with various degrees of differentiation often show various contrast patterns. Sonographers used to previously analyze and draw conclusions subjectively. However, this method lacks objective criteria and can be used only in a small area and not in a large one. Thus, the American College of Radiology proposed CEUS LI-RADS (24), which classifies the imaging outcomes based on strict criteria. A higher LI-RADS category specifies a worse prognosis (25), which was in line with the findings of the present study. In the training cohort, the proportions of patients with LI-RADS 5 and 6 were remarkably higher in the recurrence group than in the no-recurrence group. A higher LI-RADS category mostly indicates higher tumor malignancy and easier recurrence. In the present study, the LI-RADS category had a higher HR than the tumor diameter and serum AFP level, indicating that the LI-RADS category was an important independent predictive marker. However, a high LI-RADS category does not guarantee that patients will experience ER because whether there will be recurrence cannot be determined by tumor malignancy alone but by the synergistic role of age, liver function, operating level of the surgeon, and other factors.

Furthermore, the larger the tumor diameter, the more likely it is to recur postoperatively (26–28). Our findings also confirmed this, and the relative risk of recurrence increased by 13.3% for every 1 cm increase in the tumor diameter, which was similar to the findings of Zhang (29). Milan and the University of California, San Francisco standards also recommend that the tumor diameter has an impact on prognosis. Increased tumor diameter often indicates increased tumor aggressiveness, resulting in liver cancer recurrence. A larger tumor diameter suggests that the tumor cells grow faster and are more likely to cause pseudo-envelope rupture (30, 31), allowing the tumor cells to spread to the surrounding tissues and lead to tumor cell implantation. The larger the tumor diameter, the significantly longer the operation time will be (32), thus affecting the patient’s immune system and causing the body to be in a state of constant stress, further resulting in tumor recurrence (33, 34).

AFP is a routine screening marker for patients with liver cancer. In patients positive for AFP preoperatively, changes in the serum AFP level can be used to monitor the effect of surgery. A preoperative serum AFP level of >1000 ng/mL was highly associated with postoperative cancer recurrence. Thus, a high serum AFP level may be an alternative parameter of a good predictor of postoperative liver cancer recurrence (35). A higher serum AFP level often indicates that the tumor is more aggressive. The higher the preoperative serum AFP level, the greater the probability of cancer cells metastasizing through the bloodstream. Therefore, the serum AFP level plays a significant role in the identification of the tumor grade and prediction of prognosis. There is no uniform standard for the optimal cutoff value for serum AFP level to predict postoperative recurrence. In the present study, we divided the patients into three categories of serum AFP levels: 0–20, 20–200, and >200 μg/L; the HR of recurrence at each level was 1.309. Our findings are consistent with those of another study (35). HCC is often associated with elevated serum AFP levels; however, this is not the case in some patients with HCC. Moreover, mildly elevated serum AFP levels may be associated with hepatitis, pregnancy, and reproductive tumors and should be carefully considered (36).

In the preliminary statistics of this study, we also found that many other factors, such as TBIL, prothrombin time; prothrombin time activity, starting time, iso-time, and enhancement type, were predictive of postoperative recurrence. However, considering the predictive accuracy and clinical generality, our final prediction model did not include these factors; nonetheless, their exclusion does not necessarily indicate that they are not important. We found that a higher TBIL level was associated with a higher recurrence risk, which was similar to the findings of a previous study (37). A higher TBIL indicates more severe liver damage and a higher recurrence risk. A longer starting time and iso-time signify a lower recurrence risk, higher tumor differentiation, and lower recurrence rate. Moreover, a shorter starting time indicates a higher recurrence rate (38). In the present study, homogeneous enhancement was a protective factor for tumor recurrence. Homogeneous tumor enhancement indicates less internal heterogeneity, no remarkable mutations, and relatively lower malignancy. Therefore, enhancement types can also be used as an important marker for predicting recurrence.

## Limitations

This was a retrospective observational study based on a single study center and thus has some limitations that should be highlighted. Retrospective studies are necessarily subject to the risk of selection bias. CEUS requires a clear ultrasound image, and if the two-dimensional ultrasound is unsatisfactory, the CEUS image will not be suitable for further analysis. Furthermore, CEUS is very dependent on the experience of the operator. In some patients, such as those with obesity or in those in whom the tumor is located in an ultrasound-blind area (near the top of the diaphragm), the image will not be available for evaluation. Although we have validated the model using an independent internal cohort and achieved acceptable results, it has not been externally validated; therefore, its application is limited. We plan to conduct a multi-center study, expand the sample size, and perform prospective validation in the future.

## Conclusion

The noninvasive predictive model constructed in the present study has positive indicative significant implications for distinguishing patients with high-risk recurrence risk after HCC surgery. Prospective and multicenter studies need to be conducted in the future to validate whether the model is worth promoting.

## Data availability statement

The data supporting the findings of this study are available from the corresponding author [XW] on request.

## Ethics statement

This study received local ethics committee approval and Institutional Review Board approval (Approval No. 2020-010-01). All patients were informed in writing of the study protocol and objectives. The patients/participants provided their written informed consent to participate in this study.

## Author contributions

HT construct and write the article. XW guide the all process. SF, LC, YH, JZ give assist for this article. All authors contributed to the article and approved the submitted version.
